# Microbial Potential for Ecosystem N Loss Is Increased by Experimental N Deposition

**DOI:** 10.1371/journal.pone.0164531

**Published:** 2016-10-13

**Authors:** Zachary B. Freedman, Rima A. Upchurch, Donald R. Zak

**Affiliations:** 1 School of Natural Resources & Environment, University of Michigan, Ann Arbor, Michigan, United States of America; 2 Department of Ecology and Evolution, University of Michigan, Ann Arbor, Michigan, United States of America; National Taiwan University, TAIWAN

## Abstract

Fossil fuel combustion and fertilizer use has increased the amount of biologically available N entering terrestrial ecosystems. Nonetheless, our understanding of how anthropogenic N may alter the physiological mechanisms by which soil microorganisms cycle N in soil is still developing. Here, we applied shotgun metagenomics to a replicated long-term field experiment to determine how two decades of experimental N deposition, at a rate expected by mid-century, has affected the genetic potential of the soil microbial community to cycle N in soils. Experimental N deposition lead to a significant and persistent increase in functional assemblages mediating N cycle transformations associated with ecosystem N loss (*i*.*e*., denitrification and nitrification), whereas functional assemblages associated with N input and retention (*i*.*e*., N fixation and microbial N assimilation) were less positively affected. Furthermore, the abundance and composition of microbial taxa, as well as functional assemblages involved in housekeeping functions (*i*.*e*., DNA replication) were unaffected by experimental N deposition. Taken together, our results suggest that functional genes and gene pathways associated with ecosystem N loss have been favored by experimental N deposition, which may represent a genetic mechanism fostering increased N loss as anthropogenic N deposition increases in the future.

## Introduction

Anthropogenic deposition of biologically-available nitrogen (N) is projected to continue throughout this century [1, 2] and understanding the fate of anthropogenic N has a myriad of implications for the function of terrestrial ecosystems. For example, the potential health and environmental damages of anthropogenic N are estimated to be $210 billion USD yr^−1^ in the United States alone [3]. In N-limited temperate forests [4], increased N availability may lead to a phenomenon called N saturation, which predicts ecosystem responses as N limitation to plants is alleviated, resulting in greater N loss via leaching and denitrification [5, 6]. Sugar maple (*Acer saccharum* Marsh.) dominated forests in the upper Great Lakes region, U. S. A., are particularly prone to N saturation, due to their high rates of net N mineralization (80–120 kg N ha^-1^ y^-1^) and moderate rates of atmospheric N deposition (7–12 kg N ha^-1^ y^-1^ [7–9]). Anthropogenic N deposition can decrease microbial respiration and biomass [10–12], and alter bacterial and fungal community composition [13–16]. However, we have a limited understanding of whether anthropogenic N deposition can alter the abundance and composition of soil microbial communities mediating N cycling processes and whether any changes will exacerbate ecosystem N loss [17, 18].

**Fig 1:**
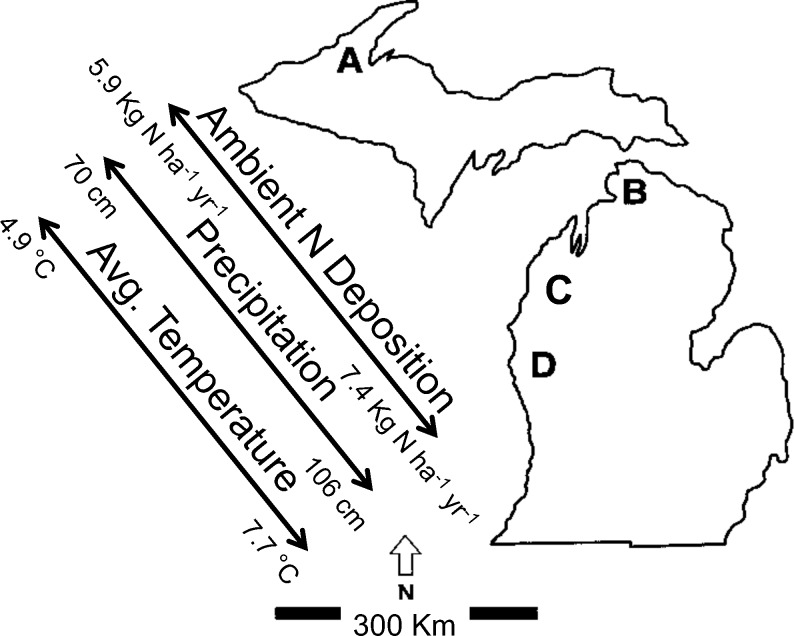
The geographic distribution of the study sites in Lower and Upper Michigan. In each stand beginning in 1994, three plots received ambient atmospheric N deposition and three plots received ambient plus 30 kg NO_3_^−^–N ha^−1^ yr^−1^.

Whole-community surveys (*e*.*g*., shotgun metagenomes) are essential to understand of microbial responses to anthropogenic N deposition, especially regarding the functional assemblages mediating soil N cycling processes. For twenty years, we have experimentally increased N deposition in replicate northern hardwood forest stands to investigate the ecosystem-level consequences of chronic anthropogenic N deposition (Table 1 and Fig 1 [1]). To date, experimental N deposition has increased NO_3_^-^ leaching and soil C storage [8, 19, 20]. It has also altered the composition of bacteria [21, 22] and fungi [23, 24] in forest floor. Furthermore, experimental N deposition altered the bacterial and fungal potential to degrade lignocellulose [16, 25, 26].

**Table 1:** Site, climatic, overstory, and ambient nitrogen deposition rates of four study sites receiving experimental N additions.

Characteristic		Site A	Site B	Site C	Site D
Location					
	Latitude (N)	46°52”	45°33”	44°23”	43°40”
	Longitude (W)	88°53”	84°52”	85°50”	86°9”
Climate					
	Mean annual temperature (°C)	4.8	6.1	6.5	7.7
	Mean annual precipitation (cm)	91.9	93.3	92.8	86.6
	Wet + Dry Ambient N Deposition (Kg N ha^−1^ yr^−1^)	6.8	9.1	11.7	11.8
Vegetation					
	Overstory biomass (Mg ha^-1^)	261	261	274	234
	*Acer saccharum* biomass (Mg ha^-1^)	237	224	216	201
Environment					
*Leaf Litter (Oe/Oa horizons)*					
	Litter C:N	63.7	57.1	52.9	43.4
	Litter mass (g)	412	396	591	550
*Soil (0–10 cm)*					
	Sand (%)	85	89	89	87
	pH (1:1 soil/H_2_O)	4.8	5.0	4.5	4.7
	Base saturation, %	71	96	73	80

In this study, we applied shotgun metagenomics to decipher whether nearly two decades of experimental N deposition has altered the functional capacity of the soil microbial community to cycle N in our long-term field experiment. Nitrogen saturation theory predicts that northern forest ecosystems will exhibit greater rates of N cycling processes (*i*.*e*., denitrification and NO_3_^-^ leaching) with reduced N limitation to plants. We hypothesized that forest stands exposed to future rates of N deposition will harbor functional genes associated with N cycle processes in a greater abundance than forest stands exposed to ambient N deposition, especially those mediating denitrification. We analyzed the shotgun metagenomes with commonly used functional gene databases (*i*.*e*., FunGene and Subsystems) to investigate the effect of experimental N deposition on the genetic capacity of soil bacteria to mediate soil N cycling processes. Using these approaches, we obtained insight to the taxonomic composition as well as the genetic capacity of the soil microbial community, which has implications to understanding the fate of anthropogenic N in soil.

## Materials and Methods

### Site description and sample collection

The influence of experimental N deposition on the N-cycling capacity of the forest floor (Oe/a horizon) microbial community was investigated in four stands of northern hardwood forest in Lower and Upper Michigan, USA (Table 1, Fig 1). The forest stands span the north-south range of the sugar maple (*Acer saccharum* Marsh) dominated northern hardwood forests in the Great Lakes region of North America [27]. All sites lie along a climatic gradient and are floristically and edaphically similar [28]. The Oi horizon is mainly comprised of sugar maple leaf litter, and the Oe/Oa horizon (*i*.*e*., forest floor) is interpenetrated by a dense mat of fine roots (~0.5 mm dia.). Soils are sandy (85–90%), well-drained, isotic, frigid Typic Haplorthods of the Kalkaska series. In 1994, six plots (30-m by 30-m) were established at each stand; three receive ambient N deposition and three receive experimental N deposition. The experimental N deposition treatment is applied during the growing season and consists of six uniform applications of NaNO_3_ pellets broadcast over the forest floor (30 kg N ha^-1^y^-1^). In our study sites, NO_3_^-^ comprises ~60% of atmospheric N deposition [20, 29]. Sampling was allowed under a special use permit issued by the USDA Forest Service and the Michigan Department of Natural Resources.

Samples from all four sites were taken during a period that was phenologically similar across the expanse of northern hardwood forest (*i*.*e*., late May to early June 2013), a time at which high rates of microbial activity are supported by ample soil moisture. Within each 30-m-by-30-m plot, 10 random 0.1-m-by-0.1-m samples of the forest floor (Oe/Oa horizons) were collected after removing the Oi horizon, composited and hand-homogenized by plot, and immediately flash frozen with liquid N_2_ for nucleic acid extraction. The forest floor was the focus of this study, as prior work has shown the microbial community inhabiting the Oe/Oa horizons to be both a sink for anthropogenic N as well as sensitive to chronic N deposition [16, 25, 30]. Samples were transported to the University of Michigan within 48 hours, where they were stored at -80°C prior to nucleic acid extraction.

### DNA extraction and metagenome generation

DNA was extracted from 0.60 g (fresh weight; six replicate extractions) of forest floor sample using a PowerLyzer PowerSoil DNA isolation kit and a PowerLyzer 24 homogenizer (MoBio Laboratories, Carlsbad, CA). The manufacturer’s protocol was followed and was further optimized by the initial addition of 250 μL phenol:chloroform:isoamyl alcohol (25:24:1; pH 6.7); bead beating for 45 seconds (4,000 rpm); all centrifugation steps at 4°C; and an overnight ethanol precipitation at -20°C. A PowerClean DNA Cleanup kit (MoBio) was used to purify the extracted DNA. Purified DNA quality was determined using a Nanodrop ND8000 (Thermo Scientific, Waltham, MA, USA) and then quantified by PicoGreen (Invitrogen, Carlsbad, CA, USA) on a Synergy HT fluorometer.

Shotgun sequencing of purified environmental DNA was performed at the University of Michigan DNA sequencing core. The sample DNA was barcoded by plot (*n* = 24) and sequenced across six lanes of an Illumina Hiseq 2500 (IIllumina, San Diego, CA, USA), with 150 base single-end reads. Cutadapt was used to remove sequences containing adapter contamination (version 1.7.1 [31]). Sequences were processed for quality control in MG-RAST [32] using default parameters and a minimum acceptable phred score of 20 in DynamicTrim [33]. Metagenome sequence data are available for public use in MG-RAST under accession numbers 4614815.3–4614838.3.

### Taxonomic identification and diversity of the forest floor metagenome

Taxonomic information was obtained from the shotgun metagenomes within MG-RAST using the “best-hit classification” against the RDP database [34]. Metagenome sequences were taxonomically assigned using a maximum e-value of 1 × 10^−5^, minimum percent identity cutoff of 80%, and a minimum alignment length cutoff of 75. Metagenomic diversity was evaluated using the alpha diversity estimator in MG-RAST.

### Identification of Functional genes

To determine the impact of experimental N deposition on the genetic potential of the forest floor microbial community to cycle N, we assessed the metagenomes as i) bacterial genes associated with N-cycle function using the FunGene database [35], and ii) functional pathways (*i*.*e*., including promoters, transport proteins, etc.) using a Subsystems-based approach in MG-RAST [36]. Inasmuch, we obtained complimentary assessments of how chronic N deposition impacted the genetic capacity of the forest floor microbial community to mediate N cycling processes.

Sequences from shotgun metagenomes were assigned to a putative function by homology to reference databases for genes associated with N cycle transformations. Reference databases were downloaded from FunGene, sequences were included that achieved a minimum score of 100 and a greater than 50% coverage to the Hidden Markov Model; the minimum score was increased for some genes to remove non-specific reference sequences from the database. (S1 Table; *sensu* [37]). FunGene databases for typical and atypical *nosZ* were combined as a single *nosZ* database to reduce the possibility of redundant annotations. A total of twelve FunGene databases for genes associated with N cycle function were used in further analyses (S1 Table). The abundance of genes associated with N cycle transformations was determined by the assignment of metagenome sequences to each functional gene database using the ‘blastx’ function in DIAMOND (v. 0.7.9.58 [38]). FunGene databases were retrieved on 6/16/2016 and are publicly available on GitHub (https://github.com/zacf/UM-gradient-metagenome-databases).

For Subsystems analysis, metagenome sequences were annotated to functional pathways using a maximum e-value of 1 × 10^−5^, a minimum percent identity cutoff of 60%, and a minimum alignment length cutoff of 25 amino acids. To gain insight in to how microbial N cycling is affected by experimental N deposition, we examined the functional pathways (*i*.*e*., Subsystem level three) within the “N cycle” level-one classification.

### Housekeeping gene identification

We assessed genes involved in cellular “housekeeping” functions (*e*.*g*., DNA repair and replication) to assess whether experimental N deposition increased the relative abundance of bacterial genes mediating soil N cycling processes. It is plausible that experimental N deposition could increase the occurrence of both housekeeping genes and those involved in mediating soil N cycling processes; or, the abundance of function genes could increase, while the abundance of housekeeping genes remained constant. We evaluated the effect of N deposition on housekeeping genes by querying the metagenomes against i) housekeeping gene databases downloaded from FunGene, including DNA gyrase (*gyrB*), DNA recombinase (*recA*), and RNA polymerase (β-subunit; *rpoB*) using DIAMOND (S1 Table), and ii) 36 gene pathways within the “DNA metabolism” Subsystems level 3 classification in MG-RAST. We used this information to assess whether experimental N deposition increased the abundance of functional genes that mediate ecosystem processes, relative to those involved with basal metabolic processes. By doing so, we could determine if experimental N deposition increased the overall prevalence of particular N cycling genes in the forest floor bacterial community.

### Statistical Analyses

For each metagenome, functional gene and gene pathway assignments were normalized to the number of reads with predicted functions (*n* = 24; *sensu* [39]). In the circumstance in which sequences were assigned to multiple Subsystem or gene categories, all categories were counted as additional hits (*sensu* [40]). All univariate analyses were performed in R (Version 3.01 [41]). The effect of experimental N deposition on the relative abundance and diversity of hits attributed to functional genes and pathways were evaluated by two-way ANOVA with site, treatment, and their interaction as factors; means were compared with a Tukey’s Honestly Significant Difference test (HSD [42]). Significance was accepted at α = 0.05; when applicable, P-values were corrected for multiple comparisons using the Benjamini & Hochberg false discovery rate correction [43].

Calculation of beta-diversity and associated statistics were performed in R and Primer (version 6, Primer-E Ltd., Plymouth, UK). The normalized metagenome abundance table was used to generated a Euclidian distance matrix, from which, the compositional differences between microbial N-cycling and housekeeping assemblages exposed to ambient and experimental N deposition were evaluated by permutational multivariate analysis of variance using the “adonis” function in R (PerMANOVA [44]). Site, treatment, and their interaction were included as factors in the two-way PerMANOVA model. Contributions of each gene or gene pathway to the metagenomic dissimilarity between the ambient and experimental N deposition condition were assessed using Similarity Percentage analysis (SIMPER [45]) in Primer.

## Results

### Taxonomic assessment of the soil microbial community

We analyzed high-quality single-end reads to determine if experimental N deposition altered the genetic capacity of the soil microbial community to cycle N in soil (*sensu* [39, 46, 47]). Across the 24 metagenomes (*i*.*e*., 12 plots receiving ambient N deposition and 12 plots receiving experimental N deposition), sequencing and quality control generated 717,933,077 high-quality reads totaling 108.4 Gbases. 26–33% of reads could be assigned to a putative function, percentages that are similar to other metagenomic surveys of soil [39, 48].

We determined the taxonomic classification of metagenome sequences using the RDP database within MG-RAST. Experimental N deposition did not affect the taxonomic composition of the soil microbial community at the phylum (PerMANOVA; *P* = 0.27), class (*P* = 0.30), or species (*P* = 0.21) level. Reads associated with Bacteria dominated the metagenomes (~98% of annotated reads); the bacterial phyla Actinobacteria (S1 Fig; ~46%;), Proteobacteria (~ 26%), and Bacteroidetes (~ 11%) were the major taxa across the metagenomes. Fungi (1.0 ± 0.6%) and Archaea (0.03 ± 0.01%) were less well represented across the metagenomes. The estimated alpha diversity of the metagenomes was not affected by experimental N deposition (S2 Fig; *P* = 0.80).

### Effects of experimental N deposition on functional genes associated with N Cycle transformations

**Fig 2:**
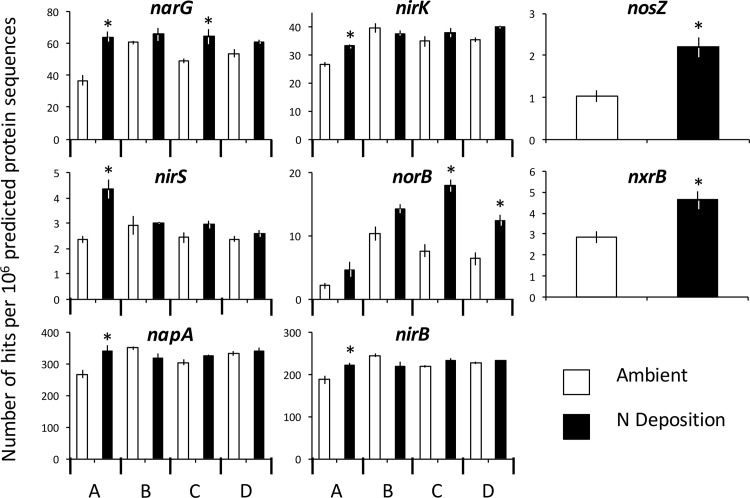
The effect of experimental N deposition on the relative abundance of genes associated with N cycle transformations. All genes presented were significantly different in abundance between the ambient (open bars) and experimental N deposition treatment (closed bars; adjusted *P* < 0.05). Data are shown by treatment unless the site × treatment *P* < 0.05, in which case the data are presented by site. Abundance data can be found in S2 and S3 Tables. * *P* < 0.05; result of pairwise tests can be found in S3 Table.

Experimental N deposition increased the abundance of functional genes in the FunGene database associated with N cycle processes, but this response mainly occurred in a site-specific manner (site × treatment; adjusted *P* < 0.05; Fig 2, S2 Table). For example, genes associated with assimilatory NO_3_^-^ reduction (*i*.*e*., *napA*, *nirB*) increased in abundance under experimental N deposition; however, this response was driven by increased gene abundances in site A, which is the northern-most site with the least amount of ambient N deposition (*napA*, +28% increase; *nirB*, +19% increase; Tukey’s HSD; *P* < 0.05). These gene assemblages were unaffected by N deposition in the southern-most three sites. Further, genes associated with denitrification increased in abundance under experimental N deposition, and again this response was driven by increased gene abundances in site A (*e*.*g*., *narG* (+74% change from ambient), *nirK* (+25%), and *nirS* (+85% change), with no change in gene abundances in the southern-most three sites. The *norB* gene, which also is associated with denitrification, increased in abundance under experimental N deposition in the southern-most two sites C (+135% increase from ambient) and D (+74% increase) and not in the northern two sites. However, the *nxrB* gene (+63% increase from ambient), which is associated with nitrification, as well as the *nosZ* gene (+111% increase), which is associated with denitrification, both increased in abundance under experimental N deposition consistently across sites (site × treatment; adjusted *P* > 0.05).

**Fig 3:**
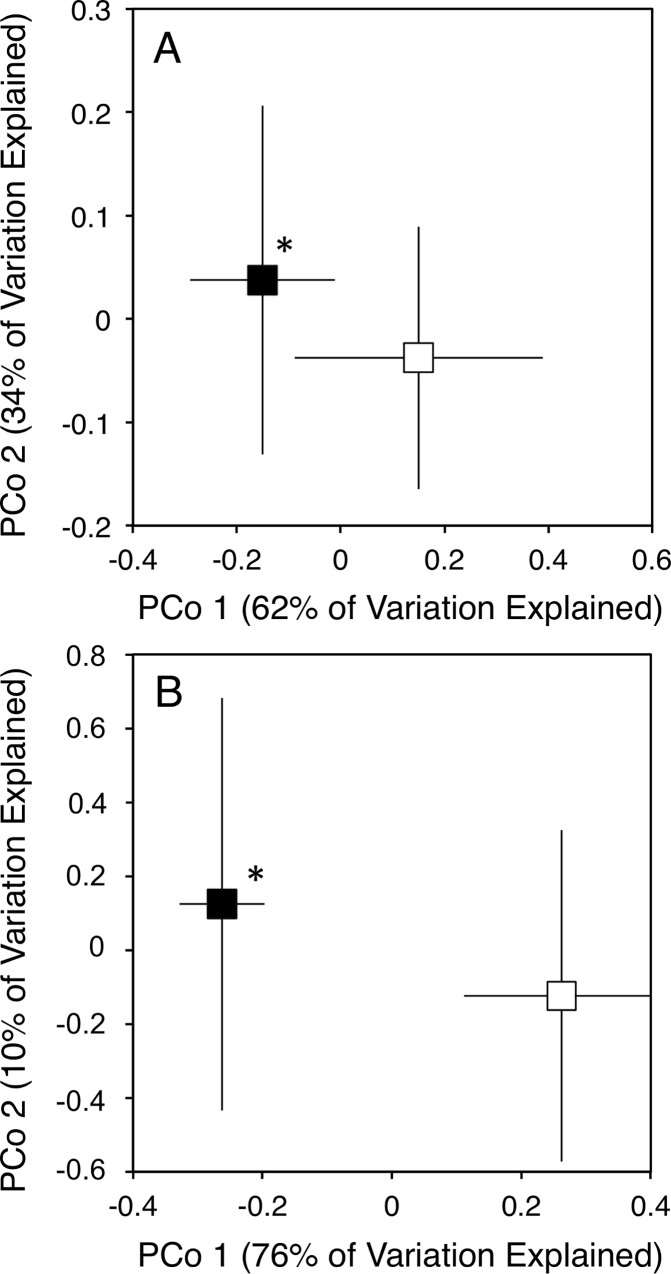
**The effect of experimental N deposition on the composition of functional genes (A) and Subsystems gene pathways (B) associated with the N cycle.** Ordinations were obtained from Principle Coordinates Analysis on based Euclidian distances of normalized data. Open and closed boxes indicate the composition of functional gene pathways in the ambient and experimental N deposition treatment, respectively. * *P* < 0.05 by PerMANOVA.

The overall composition of functional genes mediating N cycle processes was altered in a consistent manner by experimental N deposition, as there was no significant site × treatment interaction (Fig 3A; treatment effect, *P* = 0.003; site × treatment, *P* = 0.25). As indicated by SIMPER, genes associated with denitrification (Table 2; *i*.*e*., *napA* (25%), *narG* (19%)), contributed the greatest proportion of multivariate dissimilarity between the ambient and experimental N deposition treatment, followed by genes associated with assimilatory NO_3_ reduction (*i*.*e*., *nirB*, (12%) and nirA (10%)). Together, functional genes mediating denitrification and assimilatory NO_3_ reduction contributed 89% of the total dissimilarity between the microbial community under ambient and experimental N deposition.

**Table 2:** Contribution of functional genes implicated in the N cycle to compositional dissimilarity as determined by SIMPER.

Process	Gene	Average Dissimilarity	Percent Contribution	Cumulative Percent Contribution
Denitrification			67.2	67.2
	*napA*	1.8	24.9	
	*narG*	1.4	19.1	
	*norB*	1.1	15.3	
	*nosZ*	0.3	3.8	
	*nirK*	0.2	2.7	
	*nirS*	0.1	1.4	
Assimilatory NO_3_ reduction	* *		21.5	88.7
	*nirB*	0.9	12.0	
	*nirA*	0.7	9.5	
Nitrification			6.6	95.3
	*nxrB*	0.3	4.4	
	*ureA*	0.2	2.2	
N Fixation			4.8	100
	*nifD*	0.2	2.8	
	*nifH*	0.2	2.0	

### Effects of N deposition on functional pathways of the N cycle

**Fig 4:**
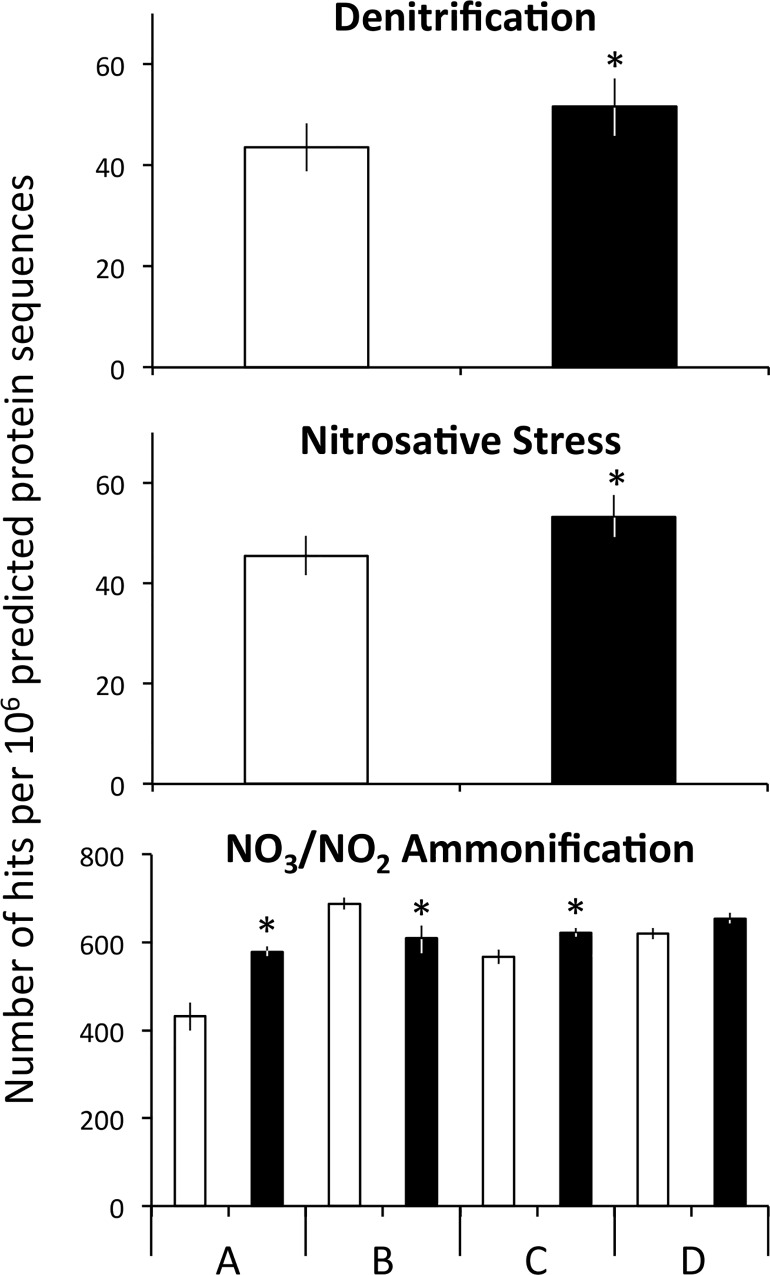
The effect of experimental N deposition on the relative abundance of Subsystems level 3 functional pathways. All pathways presented were significantly different in abundance between the ambient (open bars) and experimental N deposition treatment (closed bars; adjusted *P* < 0.05). Data are shown by treatment unless the site × treatment *P* < 0.05, in which case the data are presented by site. The full datasets can be found in S3 Table. ^**×**^ Site × Treatment; adjusted *P* < 0.05.

Experimental N deposition altered the abundance of 3 of 11 total Subsystems level 3 functional pathways within the “N Cycle” level 1 classification (Fig 4 and S4 Table; adjusted *P* < 0.05). Further, all 3 affected gene pathways increased in abundance in the experimental N deposition treatment, relative to the ambient treatment. For example, gene pathways associated with denitrification (+18% change from ambient) and nitrosative stress protection (+19%) were most positively affected by experimental N deposition, followed by gene pathways associated with NO_3_/NO_2_ ammonification (*i*.*e*., assimilatory and respiratory pathways; +9%). Gene pathways mediating nitrosative stress protection and denitrification were affected similarly by experimental N deposition (site × treatment; adjusted *P* > 0.05), however, gene pathways associated with NO_3_/NO_2_ ammonification were affected in a site-specific manner (S5 Table; site × treatment; adjusted *P* < 0.05). Pairwise tests indicate that gene pathways associated with NO_3_/NO_2_ ammonification were significantly more abundant in sites A (+34%) and C (+10%), were less abundant in site B (-11%), and were unaffected by experimental N deposition in site D.

The composition of Subsystems level 3 functional pathways was altered by experimental N deposition in a consistent fashion across all sites (Fig 3B; treatment effect, *P* = 0.04; site × treatment, *P* = 0.19). SIMPER determined that functional pathways associated with nitrilase (Table 3; 31%), denitrification (23%), and NO synthase (19%) contributed greatest to the dissimilarity in the functional pathway composition under ambient and experimental N deposition.

**Table 3:** Contribution of Subsystems pathways to compositional dissimilarity as determined by SIMPER.

Subsystems Pathway	Average Dissimilarity	Percent Contribution	Cumulative Percent Contribution Explained
Nitrilase	2.2	30.7	30.7
Denitrification	1.6	22.9	53.6
Nitric oxide synthase	1.4	18.8	72.4
Nitrosative stress protection	0.7	9.5	81.9
Nitrogen fixation	0.4	5.7	87.6

* Only those Subsystems level 3 pathways that contributed greater than 5% to the total multivariate dissimilarity was included.

### Effect of N deposition on housekeeping genes

Experimental N deposition did not affect any of the 36 Subsystems level 3 functional pathways within the “DNA metabolism” level 1 classification (S5 Table; adjusted *P* > 0.05). Experimental N deposition also did not affect the relative abundance of three functional genes associated with housekeeping functions, namely *gyrB*, *recA*, and *rpoB* (Table 4; adjusted *P* > 0.05). Similarly, experimental N deposition neither affected the composition of DNA metabolism functional pathways nor housekeeping genes (data not shown; adjusted *P* > 0.05). Thus, chronic experimental N deposition selects for functional genes and gene pathways that mediate N cycle transformations, as it did not affect genes or gene pathways involved with basal metabolic processes.

**Table 4:** The relative abundance of metagenomic hits to genes associated with “housekeeping” functions in DIAMOND.

Gene	Ambient	Experimental N Deposition
*gyrB*	486.7 ± 11.5	482.5 ± 12.1
*recA*	177.5 ± 4.3	178.3 ± 5.3
*rpoB*	1043.3 ± 15.7	1040.8 ± 25.0

Data are presented as number of hits per 1,000,000 predicted protein sequences. None were significantly different between the ambient and experimental N deposition treatment.

## Discussion

### Selection of genes associated with the N cycle under future rates of N deposition

Two decades of experimental N deposition significantly increased the abundance of functional genes and gene pathways associated with certain N cycle processes. Further, this effect was most pronounced in the northernmost sites in our long-term field experiment that have historically experienced the lowest amounts of ambient N deposition. In contrast, experimental N deposition did not affect the relative abundance, composition, or diversity of microbial taxa, as well as genes associated with housekeeping functions (*e*.*g*., DNA and protein synthesis). Consistent with N saturation theory, our findings suggest that chronic N deposition has favored a microbial community with the genetic capacity for N cycle transformations, *i*.*e*., denitrification and NO_3_/NO_2_ ammonification, which may lead to ecosystem N loss [5, 17]. However, this result differs from others who found ecological coherence in the response of microbial taxa and functional genes to N enrichment [39, 49]. Importantly, experimental N deposition in our study occurs at a rate ~10-fold lower than in other studies (30 vs. ~300 kg N ha^-1^y^-1^). It is possible that chronic N deposition, at a rate expected by mid-century across parts of eastern North America and Europe (*i*.*e*., as in our study), is sufficient to select for the presence of functional genes associated with the cycling of N, but not to cause concomitant shift in taxonomic composition. Though obtaining taxonomic data from metagenomes is not without limitations [50], a concurrent analysis of bacterial and fungal rRNA gene sequences revealed that neither were affected by experimental N deposition [24]. Taken together, these observations suggest a selection of certain genes and gene pathways mediating N cycle processes, rather than a shift in taxonomic composition that elicits a concomitant change in functional capacity. In our case, the analysis of functional genes and gene pathways, in addition to both ribosomal genes and genes associated with housekeeping functions, supports the notion that genes associated with soil N transformations are favored by experimental N deposition.

### Functional assemblages mediating ecosystem N loss are favored by experimental N deposition

In our study, complimentary metagenomic analyses (*i*.*e*., homology of metagenomic reads to FunGene databases and Subsystems functional pathways) determined that the genetic potential of the soil microbial community to cycle N has been altered by experimental N deposition, which may be one mechanism mediating ecosystem N-loss under future rates of N deposition. For example, anthropogenic N enrichment can favor ecosystem N loss in forests through increased rates of denitrification and nitrification [51, 52]. Thus, it would be expected that the abundance of functional genes and gene pathways associated with denitrification and nitrification would be greater in soils exposed to experimental N deposition as compared to the ambient N treatment. Indeed, functional gene pathways associated with denitrification increased under experimental N deposition (19% increase from the ambient treatment; Fig 4), although functional gene associated with denitrification from the FunGene database were impacted in a site-specific fashion (Fig 4; see Site-specific responses of N cycle functional assemblages to experimental N deposition). Moreover, functional assemblages associated with denitrification accounted for a significant portion of the dissimilarity between bacterial functional assemblages associated with N-cycling processes, accounting for 67% and 23% of the dissimilarity of the gene and gene pathway assemblage composition, respectively (Tables 2 and 3). The genetic potential for nitrification was also favored by experimental N deposition, as indicated by an increased abundance of *nxrB* (+63% increase from ambient). However, the rate-limiting step in nitrification is considered to be the oxidation of ammonia into nitrite, which is performed by ammonia oxidizing archaea and bacteria (AOA and AOB, respectively). The metagenomes were interrogated for the presence of ammonia-oxidizing archaea and bacteria in our experiment; however, the abundance of these functional genes was below the limit of detection of our analyses. These metagenomic changes may have ecosystem-level consequences, as a recent meta-analysis determined the abundance of genes associated with denitrification and nitrification were positively correlated with their respective process rates [17]. In our field experiment, experimental N deposition increased annual net nitrification (+27% [8]) as well as NO_3_^-^ leaching (+680% [53]), a finding consistent with other studies as well as N saturation theory [54, 55]; unfortunately process rates for denitrification in our experiment were unavailable at this time. Previous estimations indicated denitrification rates increased 5-fold with NO_3_^-^ fertilization, however, the potential for denitrification in our long-term field experiment is low since the sandy soils are well drained and highly aerobic [56]. While these findings represent a plausible genetic mechanism mediating ecosystem N loss in our field experiment, a robust analysis of microbial N cycling in the forest floor is needed to more clearly determine if the observed compositional shifts may produce a concomitant functional change in response to experimental N deposition.

Functional gene pathways associated with nitrosative stress protection increased in abundance under chronic anthropogenic N deposition (+18% change from ambient; Fig 2), which suggests an increased impact of reactive nitrogen species (*e*.*g*., NO-derived compounds; NO_x_) on an N enriched Earth. NO_x_ can arise from fossil fuel combustion as well as incomplete denitrification and nitrification (*e*.*g*., the “hole-in-the-pipe” model [57]) and has been linked to acid rain, biodiversity loss, and ozone depletion, among other environmental consequences [58–60]. In deciduous forests, NO_x_ emission increases with N enrichment and is expected to increase in the Anthropocene [61–63], although *in situ* measurements of NO_x_ emissions in our experiment were unavailable. One of the most understood mechanisms for bacterial NO detoxification involves flavohemoprotein, which enzymatically converts NO to NO_3_^-^ [64]. Genes encoding flavohemoprotein accounted for 46% of metagenome hits to the nitrosative stress protection Subsystem (data not shown), thus the increased microbial response to nitrosative stress may be one factor mediating NO_3_^-^ leaching in our long-term experiment. Microbial nitrosative stress protection may be a favored trait in the Anthropocene, and a conduit of ecosystem N loss in an N-enriched world.

### Response of functional genes mediating ecosystem N input and retention

The response of functional genes and gene pathways associated with N cycle transformations leading to ecosystem N input and retention (*i*.*e*., N fixation) were not affected by experimental N deposition in a robust fashion, which may exacerbate ecosystem N loss under future rates of N deposition. For example, functional genes and gene pathways associated with N_2_ fixation did not change in relative abundance due to experimental N deposition (Figs 2 and 4), but did account for 26% and 5% of compositional dissimilarity between functional assemblages exposed to ambient and experimental N deposition, respectively. The link between gene abundance and function for functional genes associated with N_2_ fixation remains elusive [17]. However, free-living N_2_-fixing bacteria are typically facultative in nature and can reduce N_2_ fixation as N limitation is alleviated, which can foster slowed or reduced N_2_ fixation under N enrichment [65–67].

The incorporation of N to microbial biomass and subsequently into soil organic matter is a potential a mechanism fostering N-retention as northern hardwood forests acclimate to future rates of N deposition. For example, microbial assimilation of ^15^NO_3_^-^ occurred within hours of addition in experimental N deposition plots in our long-term experiment [30]; similar findings have also been reported in lab incubations [8] and agricultural soils [68]. From this, it would be expected that functional assemblages associated with N assimilation would increase under experimental N deposition. Here, the abundance of two functional genes and a gene pathway mediating microbial N assimilation (*i*.*e*., *napA*, *nirB* and the NO_3_/NO_2_ ammonification pathway) increased under experimental N deposition, albeit in a site-dependent manner. Specifically, *napA* and *nirB* both increased in abundance in the northern-most site A (28% and 19% increase from ambient, respectively), but not in the southern-most three sites, B, C, and D. Whereas, gene pathways associated with NO_3_/NO_2_ ammonification, which includes assimilatory pathways (*e*.*g*., *NAS* and *Nar* genes), increased in abundance in sites A (+34%) and C (+10%), but decreased in abundance in site B (11% decrease from ambient) and was unaffected by N deposition in site D. However, gene pathways associated with ammonia assimilation (*e*.*g*., via glutamate dehydrogenase) were unaffected by N deposition. Together, this suggests an increased potential for nitrate and nitrite assimilation, but not ammonia assimilation under future rates of atmospheric N deposition. Nitrilase genes are associated with N assimilation in some bacteria [69, 70]. Here, gene pathways associated with nitrilase contributed most to compositional dissimilarity between functional assemblages exposed to ambient and experimental N deposition (31%; Table 3), but the abundance of this gene pathway was not affected by experimental N deposition in a significant fashion. Counter to our predictions, the genetic potential of the soil microbial community to assimilate N was not affected by experimental N deposition in a robust fashion.

### Site-specific responses of N cycle functional assemblages to experimental N deposition

The significant site by treatment interactions that were observed for functional assemblages associated with denitrification and NO_3_/NO_2_ ammonification suggests a site-specific response to experimental N deposition. The four experimental forest stands were selected to encompass the north-south range of the northern hardwood forests in the Great Lakes region of North America, which is an expansive ecosystem. Over the last decade, ambient N deposition has ranged from ~4 to ~12 kg N ha^-1^ y^-1^ across our study sites (sites A to D, respectively; Fig 1 [71, 72]). Because the N deposition treatment (30 kg N ha^-1^ y^-1^) is constant across sites, the increase in N deposition ranges from ~10-fold at site A to ~3-fold at the southern-most site D. It is possible that functional assemblages associated with denitrification and N ammonification previously exposed to relatively high ambient N deposition (*i*.*e*., the southern-most sites) may not have been impacted by our treatment in a way we would be able to detect. It is also plausible that functional assemblages associated with denitrification and N ammonification in our southernmost sites have acclimated to the experimental N deposition treatments, whereas those in the northernmost sites have not.

Previous efforts to interrogate the impact of anthropogenic N enrichment on functional genes and gene pathways associated with soil N cycling processes in our long-term field experiment determined that experimental N deposition led to a less abundant and less rich functional assemblage associated with N cycle transformations [73], which differs from results presented here. In this study, samples were taken for during late spring, whereas samples for the previous study were taken in early fall. It is plausible that differences in phenology between the spring and fall sampling times may explain the observed differences in microbial responses to chronic N deposition. For example, the microbial decomposition of fine roots is a major C source for the soil microbiome [74], and fine roots are the major source of recalcitrant plant litter in our field experiment [75]. In northern hardwood forests, rates of fine root mortality are evenly spread throughout the year, although a pulse of fine root growth occurs during spring whereas fall is a time of increased fine root mortality and loss [76]. Nitrogen cycling functional assemblages are sensitive to substrate addition (*i*.*e*., exogenous C substrates [77]), thus, seasonal differences in substrate availability from fine roots may constrain N cycling functional assemblages in our field experiment. A seasonal assessment of N cycling transformations is needed to more accurately assess the temporal robustness of the microbial response to anthropogenic N enrichment.

## Conclusion

The results presented here support the hypothesis that chronic N deposition selects for bacteria harboring functional genes and gene pathways mediating N cycling processes that are associated with ecosystem N loss (*i*.*e*., denitrification, nitrification), whereas functional assemblages associated with ecosystem N retention (*i*.*e*., N assimilation) were less positively affected by this agent of global change. The increased abundance of certain N-cycling functional assemblages occurred as part of a broad microbial response to anthropogenic N deposition which results in increased NO_3_ leaching and soil C storage, as well as reduced litter decay. Taken together, results presented here suggest that anthropogenic N deposition may fundamentally alter the potential of soil microbial communities to cycle N in soils, which is a plausible mechanism mediating increased N loss in our long-term field experiment.

## Supporting Information

S1 Fig The relative abundance of microbial phyla under ambient (white bars) and experimental N deposition (black bars) conditions. Data presented represent the mean ± SE (n = 12). “Other” includes phyla with less than 1% relative abundance, including the bacterial phyla Chlamydiae, Cyanobacteria, Chloroflexi, Spirochaetes, Tenericutes, Gemmatimonadetes, Chlorobi, Thermotogae, Deferribacteres, and Deinococcus-Thermus, and Eukaryotes Ascomycota and Basidiomycota, Bacillariophyta and Archaea Thaumarchaeota.
(TIFF)

S2 Fig The effect of experimental N deposition on the MG-RAST estimated metagenomic alpha-diversity. Data presented represent the mean ± SE (n = 12).
(TIFF)

S1 Table Number of N Cycle genes downloaded from FunGene and included in each database. (DOCX)

S2 Table The relative abundance of metagenomic hits to functional genes associated with the N cycle in DIAMOND. Data are presented as mean number ± SE (n = 12) of hits per 1,000,000 predicted protein sequences.
(DOCX)

S3 Table The relative abundance of metagenomic hits to functional genes associated with the N cycle in DIAMOND where a significant site by treatment interaction was observed. Data are presented as mean number ± SE (n = 3) of hits per 1,000,000 predicted protein sequences.
(DOCX)

S4 Table The relative abundance of metagenomic hits to Subsystem level 3 within the nitrogen metabolism level 1. Data are presented as mean number ± SE (n = 12) of hits per 1,000,000 predicted protein sequences.
(DOCX)

S5 Table The relative abundance of metagenomic hits to the DNA metabolism Subsystem level 3. Data are presented as mean number ± SE (n = 12) of hits per 1,000,000 predicted protein sequences.
(DOCX)
