# Supplementary figures and images for: Microbial Potential for Ecosystem N Loss Is Increased by Experimental N Deposition
Source: PLoS One. 2016 Oct 13;11(10):e0164531. doi: 10.1371/journal.pone.0164531 (PMC5063468; doi:10.1371/journal.pone.0164531)

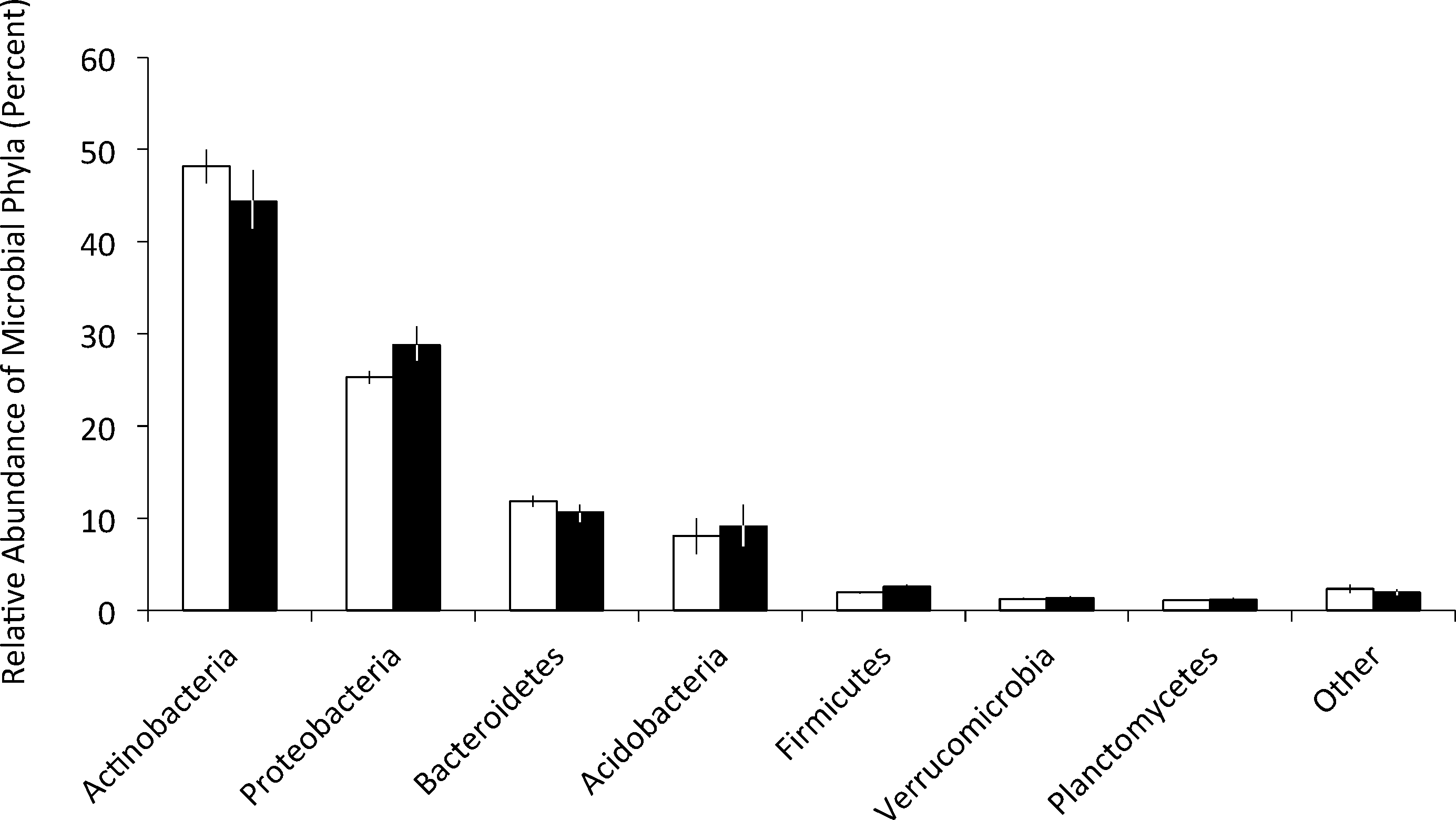

Supplement: S1 Fig — Data presented represent the mean ± SE (n = 12). “Other” includes phyla with less than 1% relative abundance, including the bacterial phyla Chlamydiae, Cyanobacteria, Chloroflexi, Spirochaetes, Tenericutes, Gemmatimonadetes, Chlorobi, Thermotogae, Deferribacteres, and Deinococcus-Thermus, and Eukaryotes Ascomycota and Basidiomycota, Bacillariophyta and Archaea Thaumarchaeota. (TIFF) [file pone.0164531.s001.tiff]

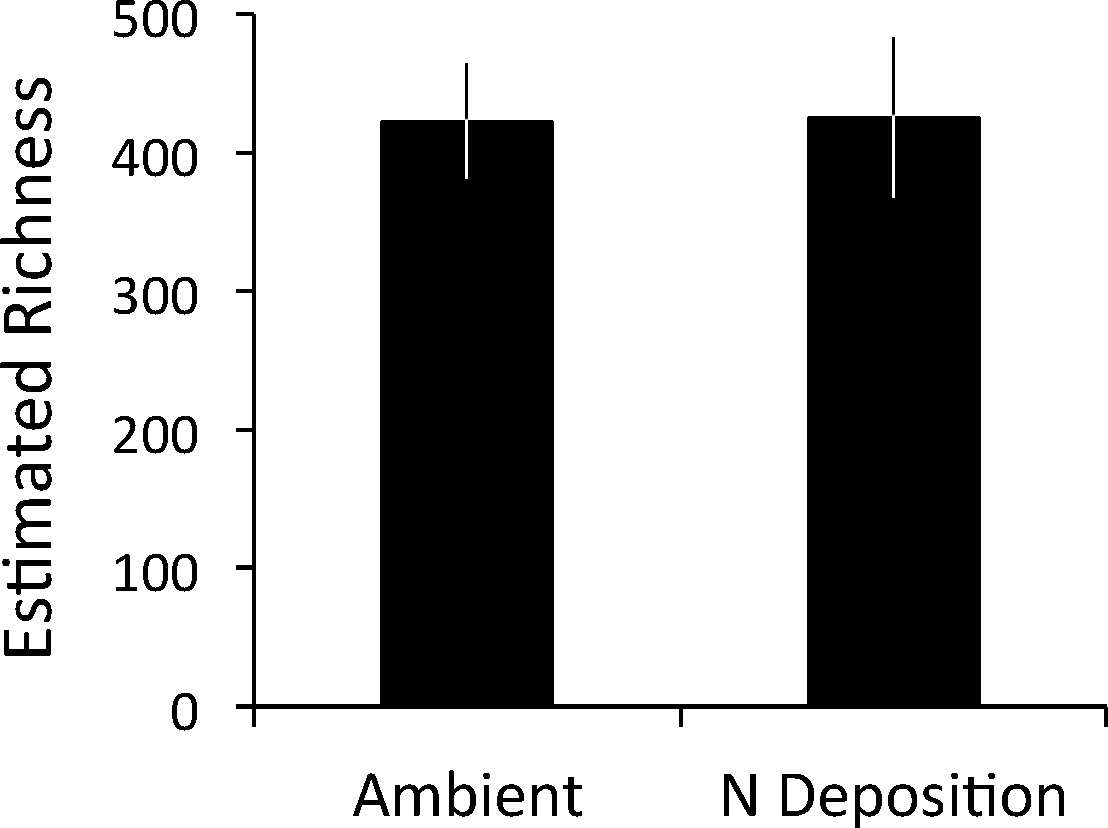

Supplement: S2 Fig — Data presented represent the mean ± SE (n = 12). (TIFF) [file pone.0164531.s002.tiff]
