# Supplementary material for: Microbial Potential for Ecosystem N Loss Is Increased by Experimental N Deposition
Source: PLoS One. 2016 Oct 13;11(10):e0164531. doi: 10.1371/journal.pone.0164531 (PMC5063468; doi:10.1371/journal.pone.0164531)
Supplement: S1 Table — (DOCX) [file pone.0164531.s003.docx]

**S1 Table. Number of N Cycle genes downloaded from FunGene and included in each database.**

| Process | Gene | All Sequences | High Quality Sequences | Min. Score for Inclusion |
| --- | --- | --- | --- | --- |
| Denitrification |  |  |  |  |
|  | *napA* | 40511 | 14522 | 305 |
|  | *narG* | 34442 | 33634 | 100 |
|  | *nirK* | 8912 | 1752 | 100 |
|  | *nirS* | 12382 | 4249 | 225 |
|  | *norB* | 7948 | 6426 | 100 |
|  | *nosZ* | 20909 | 10374 | 100 |
| N Fixation |  |  |  |  |
|  | *nifD* | 6462 | 4254 | 100 |
|  | *nifH* | 35524 | 4234 | 186 |
| Nitrification |  |  |  |  |
|  | *nxrB* | 36476 | 331 | 440^a^ |
|  | *ureA* | 42128 | 2434 | 100 |
| Assimilatory NO_3_ reduction |  |  |  |  |
|  | *nirA* | 34843 | 5260 | 488 |
|  | *nirB* | 159049 | 31170 | 258 |
| Housekeeping |  |  |  |  |
|  | *gyrB* | 112575 | 50123 | 814 |
|  | *recA* | 84702 | 69019 | 100 |
|  | *rpoB* | 98205 | 22853 | 100 |

^a^Additional nitrate reductases contaminants were removed by hand.
