# Supplementary material for: Microbial Potential for Ecosystem N Loss Is Increased by Experimental N Deposition
Source: PLoS One. 2016 Oct 13;11(10):e0164531. doi: 10.1371/journal.pone.0164531 (PMC5063468; doi:10.1371/journal.pone.0164531)
Supplement: S2 Table — Data are presented as mean number ± SE (n = 12) of hits per 1,000,000 predicted protein sequences. (DOCX) [file pone.0164531.s004.docx]

**S2 Table. The relative abundance of metagenomic hits to functional genes associated with the N cycle in DIAMOND.**

| Process | Gene | Ambient | | Experimental N Deposition | |
| --- | --- | --- | --- | --- | --- |
| Assimilatory NO_3_ Reduction | *nirA* |  | 200.9 ± 4.3 |  | 210.9 ± 3.9^×^ |
|  | *nirB* |  | 219.4 ± 6.8 |  | 226.5 ± 3.4^×^ |
| Denitrification | *napA* |  | 313.1 ± 10.2 |  | 330.7 ± 4.6^×^ |
|  | *narG* |  | 50.0 ± 2.8 |  | 63.7 ± 1.6^×^ |
|  | *nirK* |  | 34.0 ± 1.5 |  | 37.2 ± 0.9^×^ |
|  | *nirS* |  | 2.5 ± 0.1 |  | 3.2 ± 0.2^×^ |
|  | *norB* |  | 6.6 ± 1.0 |  | 12.3 ± 1.5^×^ |
|  | *nosZ* |  | 1.0 ± 0.1 |  | 2.2 ± 0.2* |
| N Fixation | *nifD* |  | 19.1 ± 0.6 |  | 19.5 ± 1.5 |
|  | *nifH* |  | 35.5 ± 0.5 |  | 35.9 ± 1.3 |
| Nitrification | *nxrB* |  | 2.8 ± 0.3 |  | 4.6 ± 0.4* |
|  | *ureA* |  | 78.2 ± 2.8 |  | 75.0 ± 2.1 |

Data are presented as mean number ± SE (n = 12) of hits per 1,000,000 predicted protein sequences.

*adjusted *P* < 0.05

^×^Site × Treatment; adjusted *P* < 0.05; pairwise data can be found in Table S5.
