# Supplementary material for: Microbial Potential for Ecosystem N Loss Is Increased by Experimental N Deposition
Source: PLoS One. 2016 Oct 13;11(10):e0164531. doi: 10.1371/journal.pone.0164531 (PMC5063468; doi:10.1371/journal.pone.0164531)
Supplement: S3 Table — Data are presented as mean number ± SE (n = 3) of hits per 1,000,000 predicted protein sequences. (DOCX) [file pone.0164531.s005.docx]

**S3 Table. The relative abundance of metagenomic hits to functional genes associated with the N cycle in DIAMOND where a significant site by treatment interaction was observed.**

|  |  | A | | B | | C | | D | |
| --- | --- | --- | --- | --- | --- | --- | --- | --- | --- |
| Process | Gene | Ambient | N Deposition | Ambient | N Deposition | Ambient | N Deposition | Ambient | N Deposition |
| Assimilatory NO_3_ Reduction | *nirA* | 184.6 ± 6.3 | 217.7 ± 17.3 | 204.9 ± 4.9 | 204.7 ± 14.4 | 198.3 ± 7.2 | 211.0 ± 5.0 | 216.0 ± 5.8 | 210.0 ± 2.0 |
|  | *nirB^x^* | 186.8 ± 10.5 | 222.4 ± 5.9* | 244.8 ± 4.9 | 218.3 ± 10.9 | 218.6 ± 1.7 | 233.1 ± 4.0 | 227.2 ± 3.4 | 232.1 ± 1.5 |
| Denitrification | *napA^x^* | 266.5 ± 12.7 | 340.8 ± 17.3* | 349.3 ± 6.9 | 317.4 ± 15.7 | 304.1 ± 8.7 | 323.8 ± 5.4 | 332.4 ± 6.5 | 340.8 ± 10.5 |
|  | *narG^x^* | 36.8 ± 3.3 | 63.9 ± 3.2* | 60.5 ± 1.4 | 65.7 ± 4.2 | 49.2 ± 1.5 | 64.1 ± 4.6* | 53.6 ± 2.6 | 61.0 ± 1.1 |
|  | *nirK^x^* | 26.5 ± 1.0 | 33.2 ± 0.6* | 39.4 ± 1.8 | 37.6 ± 1.0 | 34.7 ± 2.1 | 37.9 ± 1.5 | 35.3 ± 0.8 | 40.1 ± 0.5 |
|  | *nirS^x^* | 2.4 ± 0.1 | 4.4 ± 0.4* | 2.9 ± 0.0 | 3.0 ± 0.2 | 2.4 ± 0.2 | 3.0 ± 0.2 | 2.4 ± 0.1 | 2.6 ± 0.1 |
|  | *norB^x^* | 2.1 ± 0.5 | 4.6 ± 1.3 | 10.3 ± 1.1 | 14.1 ± 0.8 | 7.6 ± 1.0 | 17.9 ± 1.0* | 6.4 ± 1.0 | 12.4 ± 1.0* |
|  | *nosZ* | 0.5 ± 0.1 | 1.1 ± 0.4 | 1.6 ± 0.1 | 2.5 ± 0.1 | 0.9 ± 0.2 | 2.5 ± 0.6 | 1.2 ± 0.1 | 2.7 ± 0.1 |
| N Fixation | *nifD* | 21.1 ± 0.6 | 26.9 ± 3.0 | 18.9 ± 0.8 | 17.9 ± 0.8 | 18.1 ± 2.0 | 16.2 ± 0.5 | 18.4 ± 0.4 | 17.2 ± 0.5 |
|  | *nifH* | 34.9 ± 1.7 | 41.2 ± 2.9 | 35.5 ± 0.9 | 33.6 ± 2.5 | 35.4 ± 1.3 | 33.3 ± 0.5 | 36.1 ± 0.9 | 35.3 ± 0.6 |
| Nitrification | *nxrB* | 1.4 ± 0.4 | 3.5 ± 0.9 | 3.7 ± 0.3 | 5.7 ± 0.8 | 3.1 ± 0.3 | 5.0 ± 0.7 | 3.2 ± 0.3 | 4.4 ± 0.4 |
|  | *ureA* | 65.5 ± 5.3 | 65.7 ± 1.5 | 85.4 ± 0.3 | 78.3 ± 3.9 | 77.6 ± 3.8 | 73.9 ± 0.6 | 84.4 ± 1.1 | 82.0 ± 0.7 |

Data are presented as mean number ± SE (n = 3) of hits per 1,000,000 predicted protein sequences.

^x^site x treatment; adjusted *P* < 0.05

*Tukey’s HSD; *P* < 0.05
