# Supplementary material for: Microbial Potential for Ecosystem N Loss Is Increased by Experimental N Deposition
Source: PLoS One. 2016 Oct 13;11(10):e0164531. doi: 10.1371/journal.pone.0164531 (PMC5063468; doi:10.1371/journal.pone.0164531)
Supplement: S4 Table — Data are presented as mean number ± SE (n = 12) of hits per 1,000,000 predicted protein sequences. (DOCX) [file pone.0164531.s006.docx]

**S4 Table. The relative abundance of metagenomic hits to Subsystem level 3 within the nitrogen metabolism level 1.**

|  | Ambient | N Deposition |
| --- | --- | --- |
| Allantoin Utilization | 283.5 ± 19.1 | 279.2 ± 18.6* |
| Amidase^a^ | 19.7 ± 0.8 | 18.5 ± 0.6** |
| Ammonia assimilation | 2391.2 ± 106.7 | 2354.8 ± 108.5 |
| Cyanate hydrolysis | 190.3 ± 9.2 | 180.3 ± 8.0** |
| Denitrification | 43.5 ± 4.7 | 51.6 ± 5.6** |
| Dissimilatory nitrite reductase | 139.7 ± 9.8 | 143.7 ± 9.1** |
| Nitrate and nitrite ammonification | 576.8 ± 29.7 | 615.9 ± 11.3^×^ |
| Nitric oxide synthase | 504.8 ± 50.8 | 476.4 ± 48.6* |
| Nitrilase | 3.3 ± 0.4 | 3.1 ± 0.2** |
| Nitrogen fixation | 28.4 ± 2.2 | 25.3 ± 1.8^*^* |
| Nitrosative stress protection | 45.5 ± 3.9 | 53.2 ± 4.3** |

Data are presented as mean number ± SE (n = 12) of hits per 1,000,000 predicted protein sequences.

*P < 0.05

^×^Site × Treatment; P < 0.05

^a^Amidase clustered with urea and nitrile hydratase functions
