# Supplementary material for: Microbial Potential for Ecosystem N Loss Is Increased by Experimental N Deposition
Source: PLoS One. 2016 Oct 13;11(10):e0164531. doi: 10.1371/journal.pone.0164531 (PMC5063468; doi:10.1371/journal.pone.0164531)
Supplement: S5 Table — Data are presented as mean number ± SE (n = 12) of hits per 1,000,000 predicted protein sequences. (DOCX) [file pone.0164531.s007.docx]

**S5 Table. The relative abundance of metagenomic hits to the DNA metabolism Subsystem level 3.**

| Pathway | Ambient | N Deposition |
| --- | --- | --- |
| 2-phosphoglycolate salvage | 80.5 ± 2.7 | 78.0 ± 1.9 |
| ATP-dependent Nuclease | 11.5 ± 1.4 | 12.6 ± 1.5 |
| Competence in Streptococci | 0.0 ± 0.0 | 0.1 ± 0.0 |
| CRISP Cmr Cluster | 0.4 ± 0.1 | 0.3 ± 0.0 |
| CRISPRs | 33.1 ± 1.0 | 32.8 ± 1.1 |
| DNA Helicase of Unknown Function | 4.4 ± 0.6 | 3.8 ± 0.6 |
| DNA phosphorothioation | 8.2 ± 0.3 | 8.5 ± 0.3 |
| DNA processing cluster | 313.8 ± 13.8 | 311.6 ± 12.3 |
| DNA recombination, archaeal | 0.1 ± 0.0 | 0.2 ± 0.0 |
| DNA repair and recombination eukaryotic | 4.7 ± 0.6 | 5.2 ± 0.7 |
| DNA Repair Base Excision | 1999.4 ± 88.4 | 1953.6 ± 98.8 |
| DNA repair, bacterial | 1847.9 ± 78.4 | 1808.2 ± 86.0 |
| DNA repair, bacterial DinG and relatives | 127.9 ± 5.2 | 127.7 ± 1.8 |
| DNA repair, bacterial MutL-MutS system | 345.1 ± 11.8 | 335.0 ± 7.3 |
| DNA repair, bacterial photolyase | 125.2 ± 3.6 | 119.3 ± 4.5 |
| DNA repair, bacterial RecBCD pathway | 325.2 ± 23.9 | 321.5 ± 28.2 |
| DNA repair, bacterial RecFOR pathway | 852.7 ± 35.4 | 830.0 ± 39.2 |
| DNA repair, bacterial UmuCD system | 260.4 ± 7.3 | 260.9 ± 7.2 |
| DNA repair, bacterial UvrD and related helicases | 499.5 ± 19.7 | 494.3 ± 23.9 |
| DNA repair, UvrABC system | 1345.9 ± 57.5 | 1309.5 ± 65.3 |
| DNA replication, archaeal | 694.4 ± 32.1 | 684.0 ± 32.7 |
| DNA structural proteins, bacterial | 97.4 ± 2.7 | 98.2 ± 1.7 |
| DNA topoisomerases, Type I, ATP-independent | 486.1 ± 12.4 | 479.8 ± 16.1 |
| DNA topoisomerases, Type II, ATP-dependent | 1110.7 ± 41.1 | 1085.5 ± 48.8 |
| DNA-replication | 4375.8 ± 167.0 | 4294.1 ± 189.5 |
| Gram Positive Competence | 18.3 ± 0.7 | 17.6 ± 1.1 |
| Late competence | 19.5 ± 0.7 | 18.6 ± 1.1 |
| Natural DNA Transformation in Vibrio | 0.1 ± 0.0 | 0.1 ± 0.0 |
| Nonhomologous End-Joining in Bacteria | 557.7 ± 24.9 | 541.6 ± 24.5 |
| Nucleoid-associated proteins in Bacteria | 3.1 ± 0.2 | 3.2 ± 0.1 |
| Plasmid replication | 316.0 ± 10.6 | 316.6 ± 13.7 |
| Restriction-Modification System | 316.1 ± 6.5 | 323.5 ± 9.5 |
| RuvABC plus a hypothetical | 383.3 ± 14.8 | 376.9 ± 17.9 |
| Type I Restriction-Modification | 278.2 ± 5.9 | 285.6 ± 8.9 |
| Uracil-DNA glycosylase | 210.4 ± 9.5 | 207.4 ± 9.1 |
| YcfH | 88.3 ± 3.7 | 88.1 ± 3.8 |

Data are presented as mean number ± SE (n = 12) of hits per 1,000,000 predicted protein sequences.

*Adjusted *P* < 0.05
